# Smoking exposure and long-term progression of IgA nephropathy: a retrospective cohort study of 9,560 patients

**DOI:** 10.1080/0886022X.2026.2735526

**Published:** 2026-09-24

**Authors:** Wei-Bo Le, Jing-Song Shi, Feng Liu, Zhen Cheng

**Affiliations:** National Clinical Research Center of Kidney Diseases, Jinling Hospital, Nanjing University School of Medicine, Nanjing, China

**Keywords:** IgA nephropathy, smoking, kidney failure, cohort study, estimated glomerular filtration rate

## Abstract

**Background:**

Although cigarette smoking is an exposure associated with chronic kidney disease, its role in the progression of IgA nephropathy (IgAN) remains not well defined. We investigated the association between smoking exposure and adverse kidney outcomes in a large Chinese cohort of patients with IgAN.

**Methods:**

This retrospective cohort study included 9,560 adults with biopsy-proven IgAN at Jinling Hospital between 2007 and 2016. Cox proportional hazards models were used to assess kidney failure and a 50% decline in estimated glomerular filtration rate (eGFR). Because only 24 women reported smoking, the primary analysis was restricted to men and adjusted for age (using a restricted cubic spline), eGFR, log-transformed proteinuria, hypertension, diabetes, uric acid, and albumin.

**Results:**

Among 9,560 patients, 1,441 (15.1%) were current or former smokers, of whom 1,417 were men. In the 4,494 men, unadjusted hazard ratios (HRs) were 1.31 (95% confidence interval [CI], 1.13–1.51) for kidney failure and 1.26 (95% CI, 1.12–1.42) for a 50% decline in eGFR (both *p* < 0.001). After adjustment (*n* = 4,487), the corresponding HRs were 1.27 (95% CI, 1.09–1.48; *p* = 0.002) and 1.20 (95% CI, 1.07–1.36; *p* = 0.003). Estimates from the sex-adjusted full-cohort analysis were similar. No significant heterogeneity was observed across prespecified male subgroups defined by age, hypertension, eGFR, or proteinuria (all *p* for interaction > 0.05).

**Conclusions:**

Baseline current or former smoking was associated with adverse kidney outcomes among men with IgAN. Interpretation is limited by potential residual confounding and the small number of women with smoking exposure.

## GRAPHICAL ABSTRACT

**Figure:**
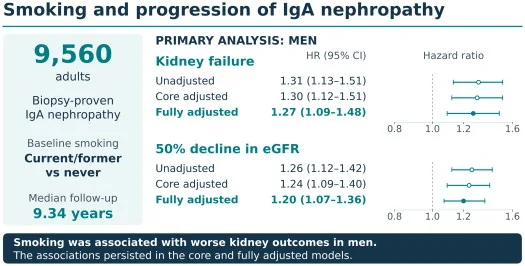

## Introduction

Chronic kidney disease (CKD) poses a substantial global health burden. Identifying modifiable factors associated with progression is therefore important. Among environmental and lifestyle factors, cigarette smoking is a well-established risk factor for cardiovascular, respiratory, and kidney diseases. Observational studies have linked smoking to CKD occurrence and progression [1–5]. However, its association with progression of immunoglobulin A (IgA) nephropathy (IgAN), the most common primary glomerulonephritis worldwide, remains incompletely characterized. IgAN is a leading cause of kidney failure in young adults, particularly in East Asian populations, with 30–40% of patients progressing to kidney failure within 20 years [6–8]. The pathogenesis of IgAN is described by the multi-hit hypothesis, involving aberrant mucosal immunity, the production of galactose-deficient IgA1, autoantibody formation, and immune complex deposition [6]. Smoking may plausibly contribute to IgAN progression through altered mucosal immunity, oxidative stress, and changes in kidney hemodynamics.

Despite this biological plausibility, clinical evidence remains limited. The Kidney Disease: Improving Global Outcomes 2025 *Clinical Practice Guideline for the Management of IgAN* recommends lifestyle modifications, including smoking cessation [9]. However, this recommendation is largely extrapolated from general CKD data rather than large-scale evidence specific to IgAN. To date, only a few retrospective studies have specifically examined the association between smoking and IgAN progression. Yamamoto et al. studied 971 Japanese patients and identified a dose-dependent association between smoking and adverse kidney outcomes, particularly among patients with lower estimated glomerular filtration rate (eGFR) [10]. Kovács et al. subsequently reported that smoking was associated with IgAN progression in a cohort of 223 patients [11]. More recently, Wang et al. analyzed 1,239 patients from West China Hospital and reported that cigarette smoking was associated with adverse kidney outcomes [12].

To evaluate the association between baseline smoking and kidney disease progression in IgAN, we conducted a retrospective cohort study of 9,560 patients with biopsy-proven IgAN. We examined whether baseline current or former smoking, compared with never smoking, was associated with kidney failure and a 50% decline in eGFR.

## Materials and methods

### Study design and population

We conducted a retrospective cohort study using the kidney biopsy registry of the National Clinical Research Center of Kidney Diseases, Jinling Hospital, Nanjing University School of Medicine, Nanjing, China. We screened patients with biopsy-proven primary IgAN diagnosed between August 2007 and August 2016. The inclusion criteria were as follows: (1) biopsy-proven primary IgAN; (2) age at biopsy ≥18 years; (3) eGFR ≥30 mL/min/1.73 m^2^ at biopsy; and (4) at least 1 year of follow-up. Patients were excluded if they had secondary IgAN attributed to systemic lupus erythematosus, Henoch-Schönlein purpura, chronic liver disease, or another autoimmune disorder, or a coexisting kidney disease such as minimal change disease, diabetic nephropathy, membranous nephropathy, or antineutrophil cytoplasmic antibody-associated vasculitis. The analysis cohort also required complete baseline smoking status, age, sex, eGFR, 24-h urinary protein, and hypertension data. Follow-up data were updated through August 2025. The study protocol was approved by the Institutional Review Board of Jinling Hospital (approval no. 2026DZKY-081-01). The study was conducted in accordance with the Declaration of Helsinki. The requirement for informed consent was waived by the Institutional Review Board because of the retrospective study design.

### Data collection and definitions

Baseline demographic and clinical data were collected at the time of kidney biopsy. Baseline smoking status was classified as never, current, or former based on the history recorded at admission. The primary exposure combined current and former smoking and compared it with never smoking; the three-category analysis in men was secondary. eGFR was calculated retrospectively using the 2021 Chronic Kidney Disease Epidemiology Collaboration creatinine equation [13]. Hypertension was defined as systolic blood pressure ≥140 mmHg, diastolic blood pressure ≥90 mmHg, or use of antihypertensive medication. Diabetes was defined by a history of diabetes or admission glycated hemoglobin ≥6.5%. Renin–angiotensin system (RAS) inhibitors, systemic glucocorticoids, and non-glucocorticoid immunosuppressants were recorded separately at baseline and during follow-up. Non-glucocorticoid immunosuppressants comprised mycophenolate mofetil, cyclophosphamide, leflunomide, cyclosporine, and tacrolimus. Baseline exposure reflected any documented use on or before biopsy, not necessarily ongoing treatment. Follow-up exposure also included sodium–glucose cotransporter 2 inhibitors and reflected prescriptions after biopsy through the registered kidney-failure event or censoring date. The primary outcome was kidney failure, defined as eGFR <15 mL/min/1.73 m^2^, dialysis initiation, kidney transplantation, or death due to kidney failure; the secondary outcome was a 50% decline in eGFR from baseline. Kidney outcomes were reviewed case by case by specialist physicians using laboratory reports and electronic medical records. Data on non-kidney death were insufficient for competing-risk analysis.

### Statistical analysis

Continuous variables were summarized as mean ± standard deviation or median (interquartile range) and compared using *t* or Wilcoxon’s rank-sum tests; categorical variables were summarized as *n* (%) and compared using *χ*^2^ or Fisher’s exact tests, as appropriate. Incidence rates per 1,000 person-years had exact Poisson 95% confidence intervals (CIs). Kaplan–Meier’s curves were compared using log-rank tests. Unadjusted and multivariable Cox models estimated smoking-associated hazard ratios (HRs) and 95% CIs. The core model adjusted for age, baseline eGFR, log-transformed 24-h proteinuria, and hypertension; the full model additionally adjusted for diabetes, uric acid, and albumin. The fully adjusted male complete-case analysis was primary; full-cohort models additionally adjusted for sex and were secondary. Age was modeled using a four-knot restricted cubic spline, with knots at the 5th, 35th, 65th, and 95th percentiles. Schoenfeld residual tests assessed proportional hazards for smoking and globally.

Sensitivity analyses separately assessed additional adjustment for body mass index (BMI), alcohol use at admission, and baseline treatment (RAS inhibitors, glucocorticoids, and non-glucocorticoid immunosuppressants). Missing binary smoking exposure (current or former vs. never) and covariates in 9,947 candidates were imputed under a missing-at-random assumption conditional on the included variables, using chained equations (50 datasets; 20 iterations), with logistic regression for binary variables and predictive mean matching for continuous variables. Imputation included the primary-model variables, both outcome indicators, and outcome-specific Nelson–Aalen cumulative hazards; estimates were pooled using Rubin’s rules. Analyses used R 4.5.3 (R Foundation, Vienna, Austria), with two-sided *p* < 0.05 considered significant.

## Results

### Baseline characteristics of the study population

As shown in Figure 1, a total of 13,925 patients with biopsy-confirmed native-kidney IgAN who met the initial biopsy and date criteria were assessed. We sequentially excluded 828 patients with concomitant other kidney diseases, including minimal change disease and other coexisting or secondary glomerular diseases, 858 with baseline eGFR <30 mL/min/1.73 m^2^, 868 aged <18 years, and 1,424 with follow-up <1 year. Of the remaining 9,947 patients, 387 with incomplete smoking status or other required baseline covariates were excluded, leaving 9,560 patients for analysis. The multiple-imputation sensitivity analysis included all 9,947 eligible candidates.

**Figure 1.**
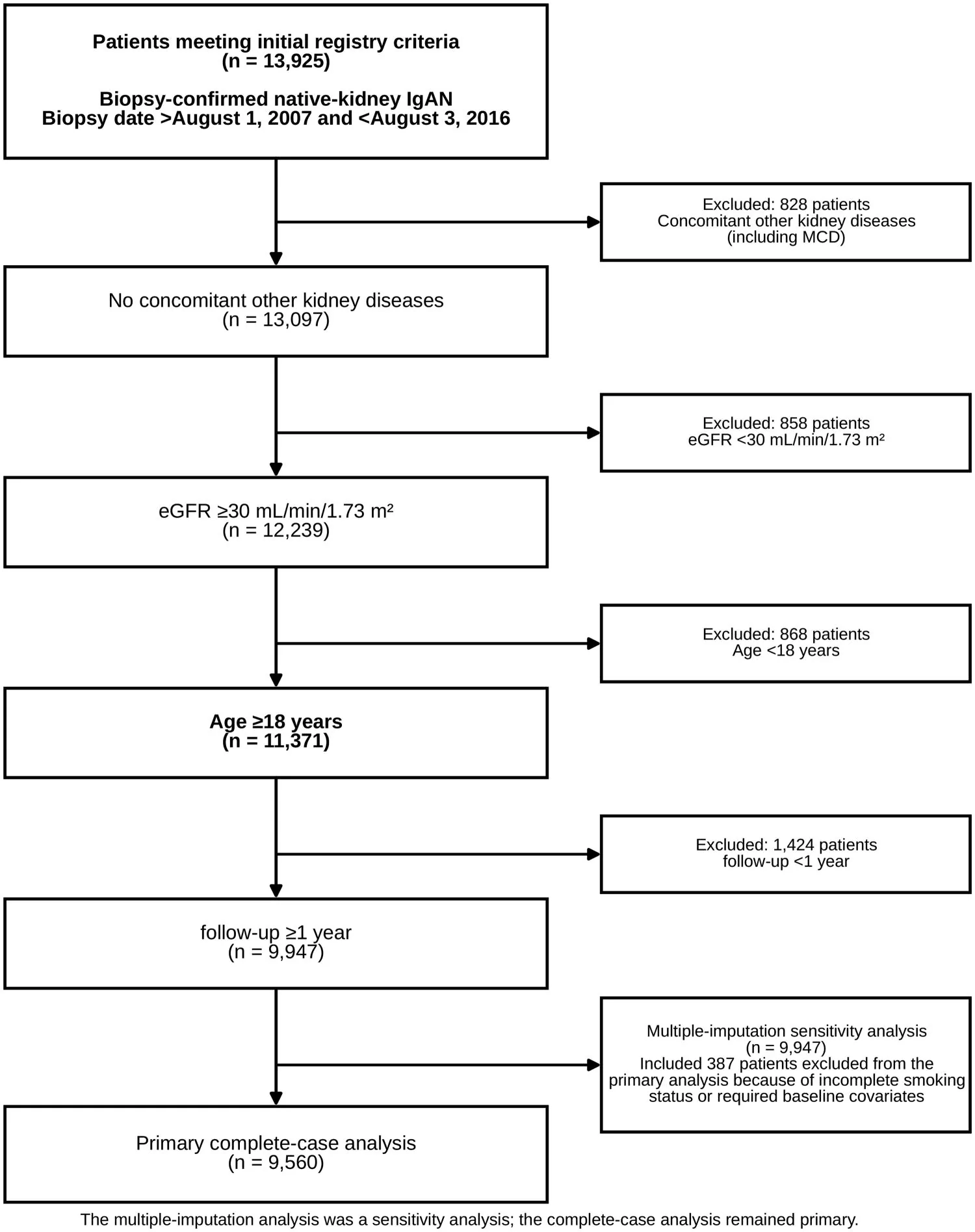
Flow diagram of cohort selection. Patients with biopsy-confirmed primary IgAN who met the initial biopsy and date criteria were sequentially screened, with exclusions for concomitant kidney diseases (including MCD and other coexisting or secondary glomerular diseases), baseline eGFR < eGFR <30 mL/min/1.73 m², age < 1 year, and follow-up < 1 year. Of 9,947 eligible candidates, 9,560 were included in the primary complete-case analysis, and all 9,947 were included in the multiple-imputation sensitivity analysis. eGFR: estimated glomerular filtration rate; IgAN: immunoglobulin A nephropathy; MCD: minimal change disease.

The baseline demographic and clinical characteristics, stratified by smoking status, are presented in Table 1. Of the total cohort, 1,441 patients (15.1%) were identified as smokers, while 8,119 (84.9%) were nonsmokers (never smokers). The smoking group comprised 1,053 current smokers (11.0% of the cohort) and 388 former smokers (4.1%). The smoking group was predominantly male (98.3% vs. 37.9% in nonsmokers, *p* < 0.001) and older at kidney biopsy (mean age 38.9 ± 10.7 vs. 35.2 ± 10.3 years, *p* < 0.001). Hypertension was present in 57.8% of smokers and 39.7% of nonsmokers, and diabetes in 6.6% and 4.2%, respectively (both *p* < 0.001). Smokers had lower eGFR (81.8 ± 26.3 vs. 93.0 ± 27.9 mL/min/1.73 m^2^, *p* < 0.001), higher proteinuria (median 1.00 vs. 0.86 g/day, *p* < 0.001), and higher serum uric acid (428.9 ± 94.6 vs. 362.9 ± 99.1 μmol/L, *p* < 0.001). Serum IgA was similar between groups (*p* = 0.853); serum immunoglobulin G was lower and complement C3 and C4 were higher among smokers. Serum albumin was higher among smokers in the full cohort (Table 1).

**Table 1.** Baseline characteristics and kidney outcomes.

	Full cohort			Men		
Characteristic	Nonsmokers (*n* = 8,119)	Smokers (*n* = 1,441)	*p*	Nonsmokers (*n* = 3,077)	Smokers (*n* = 1,417)	*p*
*Baseline characteristics*						
Age at biopsy, years	35.2 ± 10.3	38.9 ± 10.7	<0.001	34.2 ± 10.6	38.9 ± 10.7	<0.001
Male sex	3,077/8,119 (37.9%)	1,417/1,441 (98.3%)	<0.001	All men	All men	–
Body mass index, kg/m^2^	22.9 ± 3.5	24.2 ± 3.5	<0.001	23.6 ± 3.4	24.2 ± 3.5	<0.001
Hypertension	3,221/8,119 (39.7%)	833/1,441 (57.8%)	<0.001	1,531/3,077 (49.8%)	821/1,417 (57.9%)	<0.001
Diabetes	339/8,119 (4.2%)	95/1,441 (6.6%)	<0.001	127/3,077 (4.1%)	91/1,417 (6.4%)	<0.001
eGFR, mL/min/1.73 m^2^	93.0 ± 27.9	81.8 ± 26.3	<0.001	87.9 ± 27.0	81.7 ± 26.2	<0.001
24-hour urinary protein, g/day	0.86 (0.53–1.43)	1.00 (0.63–1.68)	<0.001	0.89 (0.56–1.45)	1.00 (0.64–1.68)	<0.001
Serum albumin, g/L	41.4 ± 4.9	42.1 ± 5.0	<0.001	42.7 ± 5.1	42.1 ± 5.0	<0.001
Uric acid, μmol/L	362.9 ± 99.1	428.9 ± 94.6	<0.001	422.8 ± 91.4	430.0 ± 94.0	0.015
Complement C3, g/L	0.96 (0.81–1.11)	1.00 (0.84–1.16)	<0.001	0.97 (0.83–1.13)	0.99 (0.84–1.16)	0.020
Complement C4, g/L	0.20 (0.15–0.25)	0.21 (0.17–0.27)	<0.001	0.21 (0.16–0.26)	0.21 (0.17–0.27)	0.020
Immunoglobulin A, g/L	2.76 (2.21–3.45)	2.77 (2.22–3.39)	0.853	2.88 (2.25–3.57)	2.77 (2.21–3.39)	0.002
Immunoglobulin G, g/L	11.00 (9.19–12.80)	10.40 (8.50–11.90)	<0.001	10.70 (8.89–12.40)	10.30 (8.49–11.90)	<0.001
Alcohol use at admission	469/8,115 (5.8%)	815/1,403 (58.1%)	<0.001	431/3,075 (14.0%)	809/1,380 (58.6%)	<0.001
*Treatment information*						
RAS inhibitor use at/before biopsy	3,995/6,747 (59.2%)	839/1,196 (70.2%)	<0.001	1,767/2,569 (68.8%)	827/1,176 (70.3%)	0.343
Glucocorticoid use at/before biopsy	1,126/7,193 (15.7%)	170/1,287 (13.2%)	0.025	403/2,751 (14.6%)	167/1,267 (13.2%)	0.215
Immunosuppressant use at/before biopsy	295/6,953 (4.2%)	39/1,248 (3.1%)	0.066	94/2,662 (3.5%)	37/1,228 (3.0%)	0.405
RAS inhibitor use during follow-up	6,924/8,119 (85.3%)	1,260/1,441 (87.4%)	0.032	2,728/3,077 (88.7%)	1,240/1,417 (87.5%)	0.266
SGLT2 inhibitor use during follow-up	919/8,119 (11.3%)	195/1,441 (13.5%)	0.016	436/3,077 (14.2%)	193/1,417 (13.6%)	0.622
Glucocorticoid use during follow-up	1,898/8,119 (23.4%)	278/1,441 (19.3%)	<0.001	696/3,077 (22.6%)	275/1,417 (19.4%)	0.015
Immunosuppressant use during follow-up	2,614/8,119 (32.2%)	371/1,441 (25.7%)	<0.001	896/3,077 (29.1%)	368/1,417 (26.0%)	0.029
*Follow-up and kidney outcomes*						
Follow-up duration, years	9.43 (5.22–12.31)	8.88 (4.54–11.53)	<0.001	9.38 (5.21–12.27)	8.87 (4.54–11.53)	<0.001
Kidney failure	1,141/8,119 (14.1%)	292/1,441 (20.3%)	<0.001	522/3,077 (17.0%)	287/1,417 (20.3%)	<0.001
50% decline in eGFR	1,882/8,119 (23.2%)	426/1,441 (29.6%)	<0.001	804/3,077 (26.1%)	420/1,417 (29.6%)	<0.001
Kidney failure: person-years	72,533.4	12,010.2	–	27,387.5	11,800.5	–
Kidney failure: incidence per 1,000 person-years (95% CI)	15.73 (14.83–16.67)	24.31 (21.60–27.27)	–	19.06 (17.46–20.77)	24.32 (21.59–27.30)	–
50% decline in eGFR: person-years	69,493.8	11,352.8	–	26,231.5	11,162.7	–
50% decline in eGFR: incidence per 1,000 person-years (95% CI)	27.08 (25.87–28.33)	37.52 (34.04–41.26)	–	30.65 (28.57–32.84)	37.63 (34.11–41.40)	–

CI: confidence interval; eGFR: estimated glomerular filtration rate; IQR: interquartile range; SD: standard deviation; RAS: renin–angiotensin system; SGLT2: sodium–glucose cotransporter 2.

Smoking refers to current or former smoking; the reference group is never smokers. Values are presented as mean ± SD, median (IQR), or *n*/available *N* (%). Denominators reflect available data for each variable; missing values were not imputed. *p* Values for outcome comparisons are from log-rank tests. Only 24 women were current or former smokers, so sex-specific estimates for women are unreliable. Person-years represent outcome-specific time at risk in the study cohort and may differ from the complete-case model samples. Incidence rates are expressed as events per 1,000 person-years with exact Poisson 95% confidence intervals; no separate *p* values for rate comparisons are reported. Glucocorticoids are reported separately from other immunosuppressants, which include mycophenolate mofetil, cyclophosphamide, leflunomide, cyclosporine, and tacrolimus. These definitions apply to both baseline and follow-up use. Follow-up use refers to any recorded prescription after biopsy through the kidney-failure event or censoring date. *p* Values for treatment-group comparisons are from Pearson’s chi-square tests.

Smoking exposure was highly concentrated among men: 1,417 of the 1,441 smokers (98.3%) were men, whereas only 24 women were smokers. Because this marked imbalance limited reliable inference among women, subsequent primary analyses were restricted to men, with sex-adjusted analyses in the full cohort as secondary. We therefore additionally examined baseline characteristics by smoking status among the 4,494 men. Compared with male nonsmokers (*n* = 3,077), male smokers (*n* = 1,417) were older (38.9 ± 10.7 vs. 34.2 ± 10.6 years), had a higher BMI (24.2 ± 3.5 vs. 23.6 ± 3.4 kg/m^2^), and more frequently had hypertension (57.9% vs. 49.8%) and diabetes (6.4% vs. 4.1%; all *p* < 0.001). Male smokers also had lower baseline eGFR (81.7 ± 26.2 vs. 87.9 ± 27.0 mL/min/1.73 m^2^), higher proteinuria (median, 1.00 vs. 0.89 g/day), and lower serum albumin (42.1 ± 5.0 vs. 42.7 ± 5.1 g/L; all *p* < 0.001). Serum uric acid was modestly higher among male smokers (430.0 ± 94.0 vs. 422.8 ± 91.4 μmol/L; *p* = 0.015) (Table 1).

### Kidney outcomes

During a median follow-up of 8.88 years for smokers and 9.43 years for never smokers, kidney failure occurred in 292/1,441 smokers (20.3%) and 1,141/8,119 never smokers (14.1%); a 50% decline in eGFR occurred in 426/1,441 (29.6%) and 1,882/8,119 (23.2%), respectively. Among men, kidney-failure follow-up comprised 11,800.5 person-years in smokers and 27,387.5 person-years in never smokers, with incidence rates of 24.32 and 19.06 per 1,000 person-years, respectively; corresponding follow-up for a 50% decline in eGFR was 11,162.7 and 26,231.5 person-years, with rates of 37.63 and 30.65 per 1,000 person-years. In the full cohort, kidney-failure follow-up comprised 12,010.2 and 72,533.4 person-years, with rates of 24.31 and 15.73 per 1,000 person-years; corresponding follow-up for a 50% decline in eGFR was 11,352.8 and 69,493.8 person-years, with rates of 37.52 and 27.08 per 1,000 person-years. Exact Poisson 95% CIs are reported in Table 1.

Kaplan–Meier’s analysis was used to compare event-free survival by smoking exposure. As shown in Figure 2 (men, panels A and C; full cohort, panels B and D), current or former smokers had lower event-free survival than never smokers for both kidney failure and a 50% decline in eGFR, in men and in the full cohort (all log-rank *p* < 0.001).

**Figure 2.**
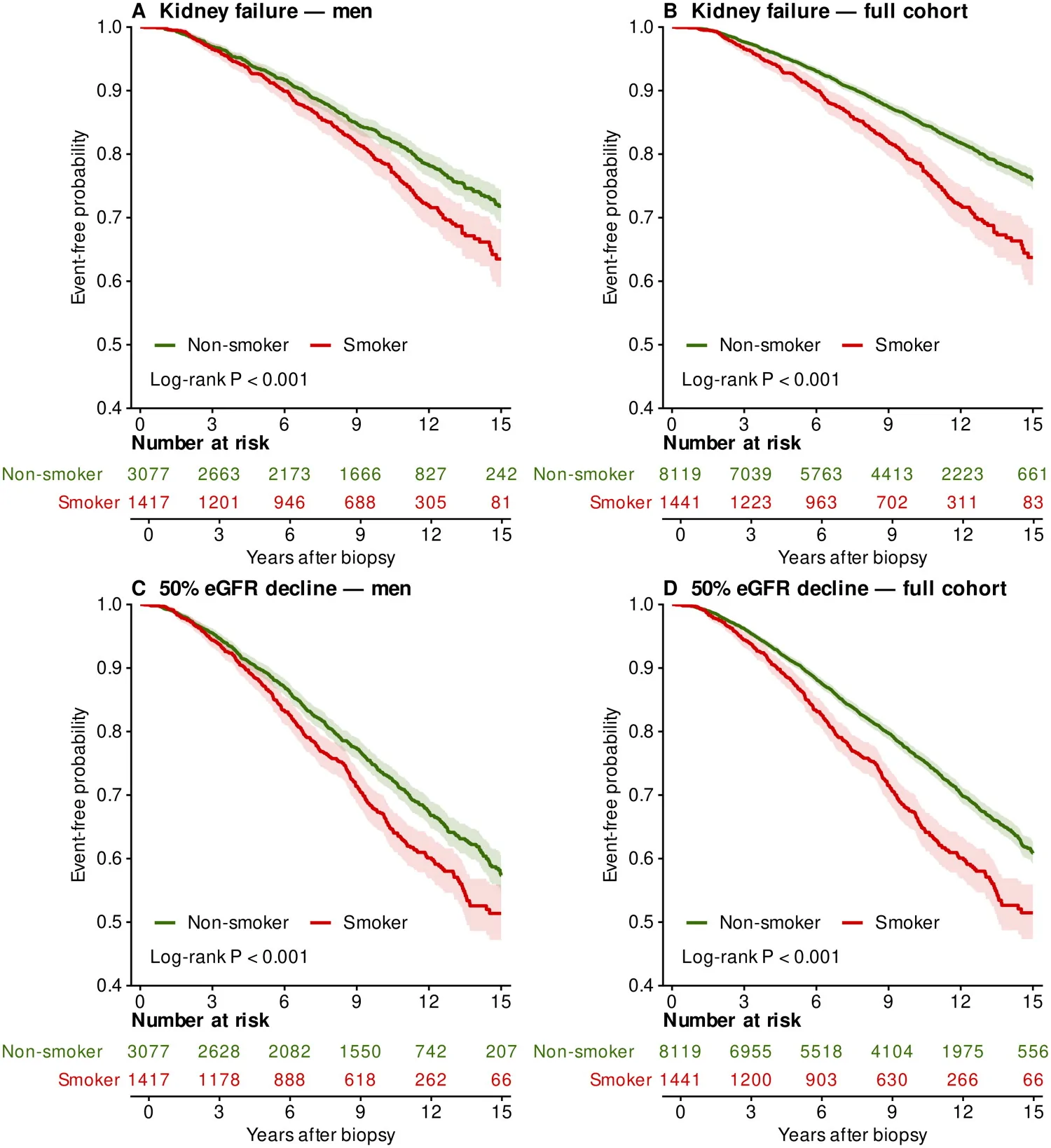
Kaplan–Meier’s analyses of kidney outcomes. Panels A and C show men (*n* = 4,494); panels B and D show the full cohort (*n* = 9,560). Smoking includes current or former smoking; never smoking is the reference group. Numbers at risk are shown below each panel. All log-rank *p* values are <0.001. Shaded bands indicate 95% confidence intervals. eGFR: estimated glomerular filtration rate.

### Association of smoking with disease progression

We used Cox proportional hazards models to evaluate the association between smoking exposure and adverse kidney outcomes (Figure 3; Table 2). Among men, the unadjusted model yielded an HR of 1.31 (95% CI 1.13–1.51; *p* < 0.001) for kidney failure and 1.26 (95% CI 1.12–1.42; *p* < 0.001) for a 50% decline in eGFR (809 and 1,224 events among 4,494 men, respectively). After core adjustment, the corresponding HRs were 1.30 (95% CI 1.12–1.51; *p* < 0.001) and 1.24 (95% CI 1.09–1.40; *p* < 0.001). In the fully adjusted model, smoking remained associated with kidney failure (HR 1.27, 95% CI 1.09–1.48; *p* = 0.002) and a 50% decline in eGFR (HR 1.20, 95% CI 1.07–1.36; *p* = 0.003), based on 4,487 men with 808 and 1,223 events, respectively. In the full cohort, the unadjusted HRs were 1.59 (95% CI 1.40–1.81; *p* < 0.001) for kidney failure and 1.43 (95% CI 1.29–1.59; *p* < 0.001) for a 50% decline in eGFR (1,433 and 2,308 events among 9,560 patients, respectively). Core adjustment plus sex attenuated the estimates to 1.31 (95% CI 1.13–1.51; *p* < 0.001) and 1.24 (95% CI 1.10–1.40; *p* < 0.001), respectively. The fully adjusted secondary model plus sex yielded HRs of 1.28 (95% CI 1.11–1.48; *p* < 0.001) and 1.21 (95% CI 1.08–1.37; *p* = 0.002), based on 9,545 patients with 1,432 and 2,306 events, respectively.

**Figure 3.**
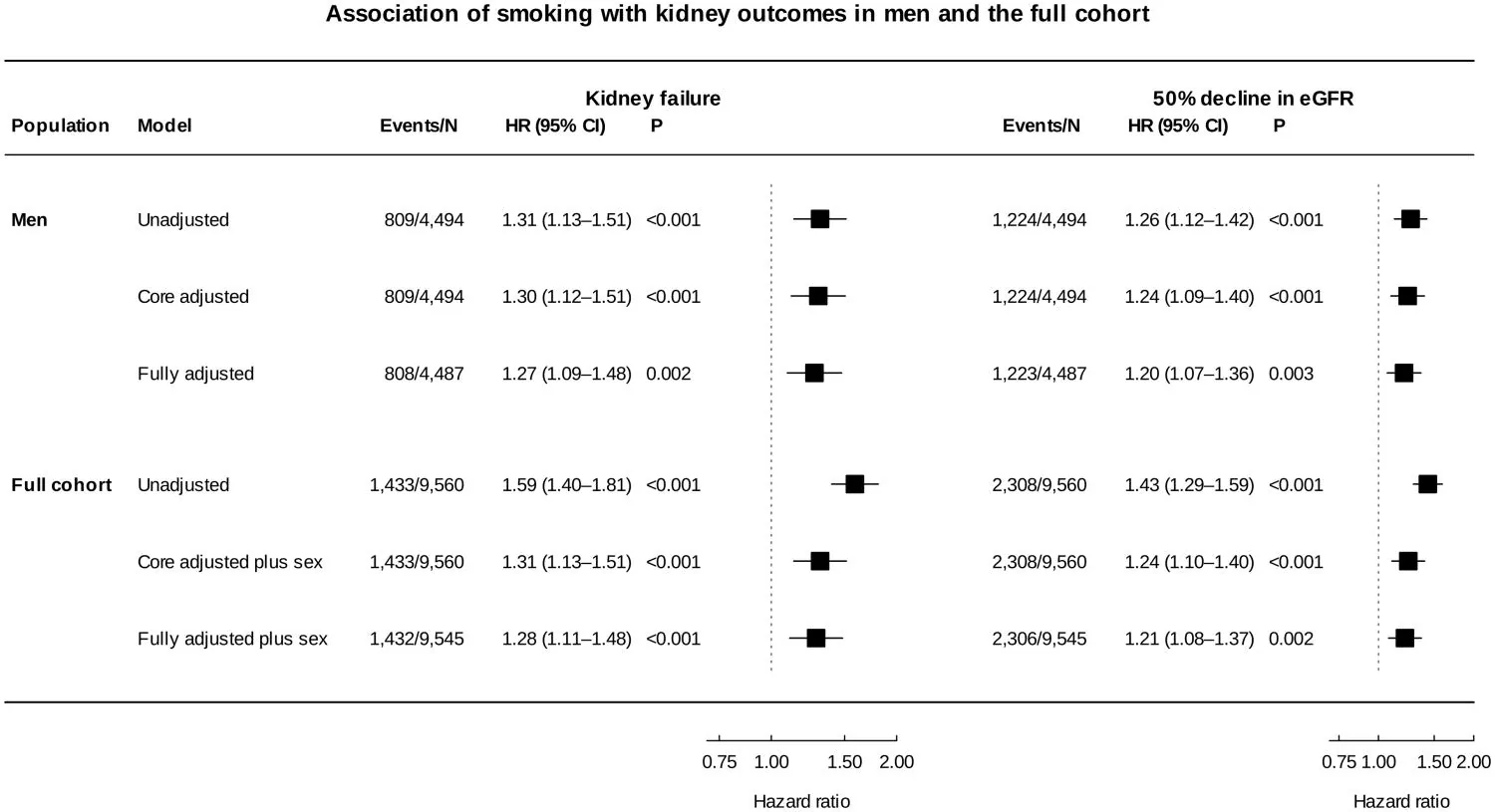
Association of smoking with kidney outcomes in men and the full cohort. The unadjusted models include smoking alone. Core adjustment in men includes smoking, a four-knot restricted cubic spline for age, baseline eGFR, log-transformed proteinuria, and hypertension; the full-cohort core model additionally includes sex. Fully adjusted models further include diabetes, uric acid, and albumin, with sex retained in the full cohort. The fully adjusted male model is the primary analysis. All model rows use regular-weight text and equal-sized filled squares. Kidney failure and a 50% decline in eGFR are shown in parallel. Events/*N* indicates the number of events relative to the sample size for each model. Horizontal lines represent 95% confidence intervals. CI: confidence interval; eGFR: estimated glomerular filtration rate; HR: hazard ratio.

**Table 2.** Association of baseline smoking with kidney outcomes.

Variable		Men, unadjusted		Men, fully adjusted		Full cohort, fully adjusted	
	Events/N	HR (95% CI)	*p*	HR (95% CI)	*p*	HR (95% CI)	*p*
*Kidney failure*							
Smoking (current or former vs. never)	809/4,494	1.31 (1.13–1.51)	<0.001	1.27 (1.09–1.48)	0.002	1.28 (1.11–1.48)	<0.001
Age (four-knot restricted cubic spline)	809/4,494	–	0.320	–	<0.001	–	<0.001
eGFR (per 10 mL/min/1.73 m^2^ higher)	809/4,494	0.65 (0.63–0.67)	<0.001	0.67 (0.65–0.70)	<0.001	0.67 (0.66–0.69)	<0.001
Log-transformed proteinuria (per 1-unit higher)	809/4,494	2.16 (1.98–2.35)	<0.001	1.36 (1.22–1.51)	<0.001	1.43 (1.32–1.55)	<0.001
Hypertension (yes vs. no)	809/4,494	2.14 (1.84–2.50)	<0.001	1.33 (1.13–1.56)	<0.001	1.50 (1.33–1.68)	<0.001
Diabetes (yes vs. no)	809/4,494	0.90 (0.65–1.24)	0.509	0.90 (0.65–1.25)	0.535	0.83 (0.64–1.08)	0.164
Uric acid (per 100 μmol/L higher)	809/4,490	1.80 (1.69–1.92)	<0.001	1.22 (1.13–1.31)	<0.001	1.24 (1.17–1.31)	<0.001
Albumin (per 1 g/L higher)	808/4,489	0.91 (0.90–0.92)	<0.001	0.95 (0.94–0.97)	<0.001	0.95 (0.94–0.96)	<0.001
Sex (female vs. male)	–	–	–	–	–	1.14 (1.00–1.29)	0.046
*50% decline in eGFR*							
Smoking (current or former vs. never)	1,224/4,494	1.26 (1.12–1.42)	<0.001	1.20 (1.07–1.36)	0.003	1.21 (1.08–1.37)	0.002
Age (four-knot restricted cubic spline)	1,224/4,494	–	0.062	–	<0.001	–	<0.001
eGFR (per 10 mL/min/1.73 m^2^ higher)	1,224/4,494	0.73 (0.72–0.75)	<0.001	0.77 (0.75–0.79)	<0.001	0.77 (0.76–0.79)	<0.001
Log-transformed proteinuria (per 1-unit higher)	1,224/4,494	2.00 (1.87–2.15)	<0.001	1.39 (1.28–1.52)	<0.001	1.42 (1.34–1.52)	<0.001
Hypertension (yes vs. no)	1,224/4,494	1.95 (1.73–2.20)	<0.001	1.38 (1.22–1.57)	<0.001	1.48 (1.35–1.63)	<0.001
Diabetes (yes vs. no)	1,224/4,494	1.09 (0.85–1.39)	0.495	1.07 (0.84–1.38)	0.575	0.94 (0.77–1.14)	0.525
Uric acid (per 100 μmol/L higher)	1,224/4,490	1.68 (1.60–1.77)	<0.001	1.26 (1.18–1.34)	<0.001	1.26 (1.20–1.32)	<0.001
Albumin (per 1 g/L higher)	1,223/4,489	0.92 (0.91–0.93)	<0.001	0.96 (0.95–0.97)	<0.001	0.96 (0.95–0.97)	<0.001
Sex (female vs. male)	–	–	–	–	–	1.26 (1.14–1.39)	<0.001

CI: confidence interval; eGFR: estimated glomerular filtration rate; HR: hazard ratio.

Smoking refers to current or former smoking; never smoking is the reference group. The fully adjusted male model includes age, baseline eGFR, log-transformed proteinuria, hypertension, diabetes, uric acid, and albumin. The corresponding full-cohort model additionally adjusts for sex. Age was modeled using a four-knot restricted cubic spline (knots at the 5th, 35th, 65th, and 95th percentiles); the *p* value represents the overall test for the age term, and no single HR is reported. A linear age specification was examined in sensitivity analyses. Units and contrasts are shown in the row labels. Adjusted-model event counts were 808 events among 4,487 men and 1,223 events among 4,487 men for kidney failure and 50% decline in eGFR, respectively; corresponding full-cohort counts were 1,432 and 2,306 events among 9,545 patients for kidney failure and 50% decline in eGFR, respectively.

When smoking status was categorized as never, current, or former among men, current and former smoking were associated with kidney failure compared with never smoking (HR 1.24, 95% CI 1.05–1.46; *p* = 0.010 and HR 1.37, 95% CI 1.06–1.77; *p* = 0.014, respectively; global exposure test *p* = 0.007). For a 50% decline in eGFR, the corresponding estimates were HR 1.21 (95% CI 1.06–1.39; *p* = 0.004) and HR 1.17 (95% CI 0.94–1.45; *p* = 0.149), with a global exposure-test *p* = 0.013. Direct comparisons of current with former smoking were not statistically significant for kidney failure (*p* = 0.463) or a 50% decline in eGFR (*p* = 0.758). These baseline-category comparisons do not establish how risk changes after smoking cessation.

### Subgroup and sensitivity analyses

In prespecified analyses among men, there was no statistical evidence that the smoking association differed by age (<40 or ≥40 years), hypertension, baseline eGFR (<60 or ≥60 mL/min/1.73 m^2^), or proteinuria (<1.0 or ≥1.0 g/day) for either outcome (all interaction *p* > 0.05; Figure 4). Confidence intervals were wide in several strata. Because only 24 women were current or former smokers, these male subgroup findings cannot be extrapolated to women.

**Figure 4.**
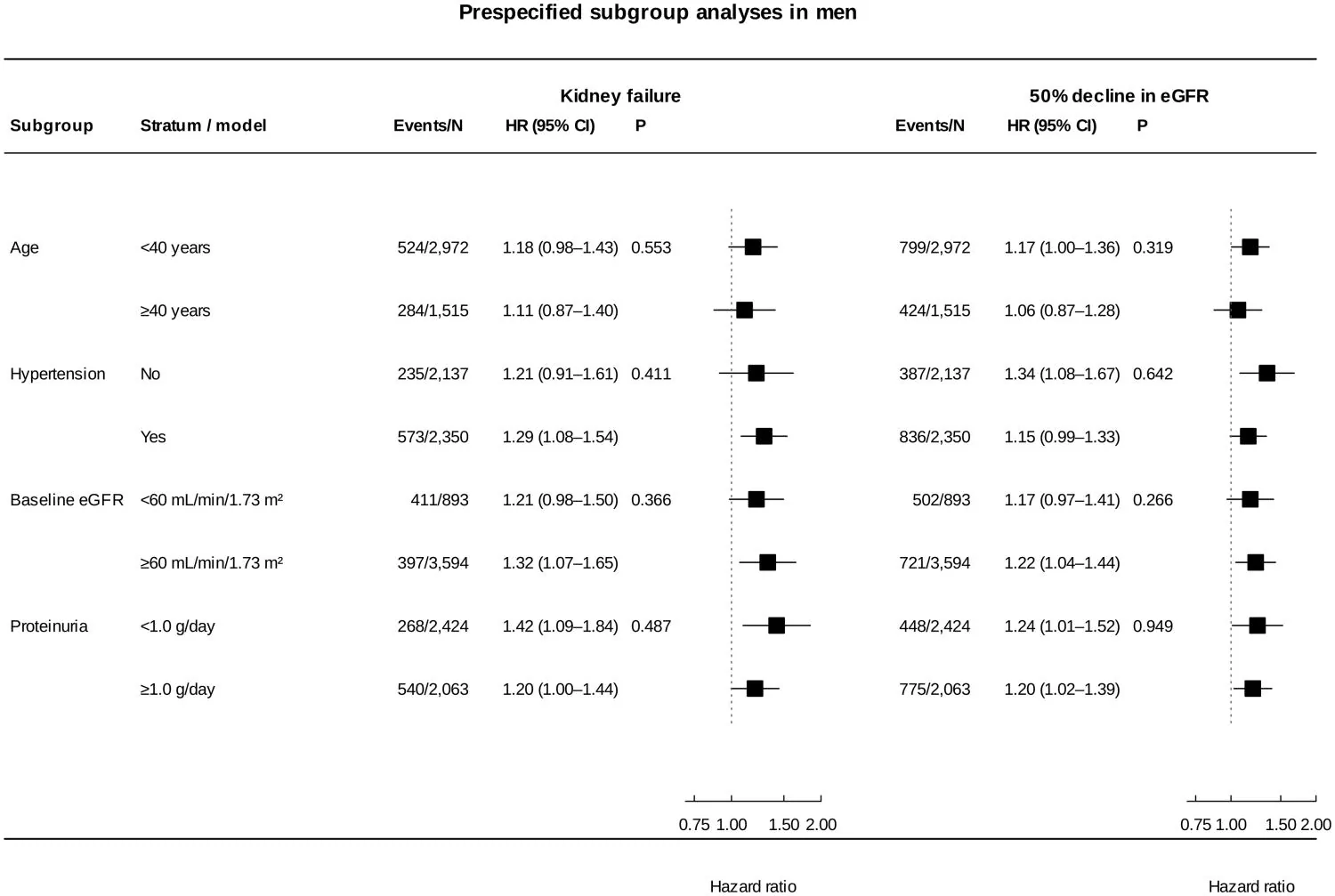
Prespecified subgroup analyses in men. Prespecified subgroup analyses are shown for age, hypertension, baseline eGFR (<60 or ≥60 mL/min/1.73 m^2^), and proteinuria (<1.0 or ≥1.0 g/day). Subgroup models omit the covariate defining the stratum and otherwise use the fully adjusted male covariates. The *p* column reports the likelihood-ratio interaction *p* value in the first row of each subgroup. Small filled squares identify subgroup estimates. Kidney failure and a 50% decline in eGFR are shown in parallel. Events/*N* indicates the number of events relative to the sample size for each model; horizontal lines represent 95% confidence intervals. CI: confidence interval; eGFR: estimated glomerular filtration rate; HR: hazard ratio.

Schoenfeld residual tests showed no evidence of non-proportional hazards for the smoking term (*p* = 0.314 and *p* = 0.494, respectively), whereas the global tests were significant for both outcomes (both *p* < 0.001), indicating non-proportional hazards for at least one adjustment covariate. In 4,434 men with complete BMI data, adding BMI to the fully adjusted model yielded HRs of 1.26 (95% CI 1.08–1.46) for kidney failure and 1.20 (95% CI 1.06–1.36) for a 50% decline in eGFR. Among 3,491 men with complete baseline treatment information, smoking HRs before and after adding RAS inhibitor, glucocorticoid, and non-glucocorticoid immunosuppressant indicators were 1.22 (95% CI 1.02–1.46) and 1.22 (95% CI 1.02–1.46) for kidney failure, and 1.17 (95% CI 1.01–1.35) and 1.16 (95% CI 1.01–1.35) for a 50% decline in eGFR. Both models used the same restricted sample. These analyses are limited by missing treatment data and confounding by indication; follow-up treatment was summarized descriptively and was not included in regression adjustment. In the multiple-imputation sensitivity analysis of all 9,947 eligible candidates (4,658 men), the pooled smoking HRs in men were 1.25 (95% CI 1.08–1.46; *p* = 0.003; 844 events) for kidney failure and 1.20 (95% CI 1.06–1.35; *p* = 0.003; 1,273 events) for a 50% decline in eGFR. Corresponding full-cohort estimates were 1.26 (95% CI 1.09–1.46; *p* = 0.002; 1,501 events) and 1.20 (95% CI 1.07–1.36; *p* = 0.002; 2,401 events), respectively.

Among 4,448 men with complete alcohol data (798 kidney-failure events and 1,211 events of a 50% decline in eGFR), smoking HRs before and after additional alcohol adjustment were 1.27 (95% CI 1.09–1.48) and 1.34 (95% CI 1.13–1.59) for kidney failure, and 1.22 (95% CI 1.07–1.38) and 1.27 (95% CI 1.11–1.46) for a 50% decline in eGFR, respectively (both alcohol-adjusted *p* < 0.001). In the full-cohort analysis with complete alcohol data (*n* = 9,503), alcohol-adjusted smoking HRs were 1.35 (95% CI 1.15–1.60) and 1.28 (95% CI 1.12–1.47), respectively (both *p* < 0.001).

## Discussion

In this retrospective cohort study involving 9,560 patients with IgAN, baseline current or former smoking was associated with higher hazards of kidney failure and a 50% decline in eGFR. In the primary analysis in men, the fully adjusted HRs were 1.27 (95% CI 1.09–1.48) and 1.20 (95% CI 1.07–1.36), respectively. Similar estimates were observed in the secondary full-cohort analysis. These observational associations are consistent with guideline recommendations for smoking cessation [9], but do not establish that smoking causes IgAN progression or that cessation improves kidney outcomes. Our findings are consistent with smaller cohorts. Yamamoto et al. reported a dose-dependent association with kidney-function decline in 971 Japanese patients [10]. Imai et al. also identified smoking as a factor associated with long-term kidney failure [14]. Wang et al. reported an association between smoking and adverse kidney outcomes in 1,239 Chinese patients [12].

Subgroup analyses found no evidence of interaction by age, hypertension, baseline eGFR, or proteinuria. However, several CIs were wide, so these findings do not establish equal associations across clinical strata. Direct comparisons between current and former smokers were also not statistically significant for either outcome. Differences in their estimates relative to never smokers therefore should not be interpreted as evidence that risks differ between current and former smokers. Without cessation timing, pack-years, or changes in smoking during follow-up, these baseline comparisons cannot establish how kidney risk changes after quitting. Differences in cumulative exposure and reasons for cessation may also influence the observed estimates. Without quantitative smoking data, this study can neither replicate nor refute the dose-dependent association reported by Yamamoto et al. nor test a dose–response relationship for the proposed biological mechanisms.

The mucosal origin of pathogenic IgA provides a biological context for the association between smoking and IgAN progression. In the multi-hit model, dysregulated immune responses at respiratory and intestinal mucosal sites contribute to galactose-deficient IgA1 production and the subsequent formation and deposition of IgA-containing immune complexes. Altered B-cell activation and homing, together with a proliferation-inducing ligand and B-cell activating factor signaling, may link mucosal immune responses to systemic production of pathogenic IgA [15]. Recent work on the intestinal microbiome–B-cell–IgA axis further implicates microbial stimulation and dysbiosis in abnormal IgA responses [16]. These pathways may complement nicotine-related mechanisms of kidney injury [17]. In murine IgAN, Kano et al. identified nasal-associated lymphoid tissue as a major induction site for nephritogenic IgA and linked Toll-like receptor 9 signaling to IgA and immunoglobulin G–IgA immune-complex formation [18]. Whether smoking disrupts these mucosal pathways in human IgAN remains a hypothesis. Nicotine-related oxidative stress and vascular injury offer complementary explanations. Jain and Jaimes reviewed the role of nicotine signaling in oxidative stress and kidney injury [17]. Clinical and histological observations in IgAN are consistent with this possibility: Myllymäki et al. reported cardiovascular and cerebrovascular disease and an association with smoking [19]; Cha et al. linked smoking to dose-dependent arteriolar hyalinosis and global glomerulosclerosis [20]; and Shinzato et al. associated smoking combined with hyperuricemia with greater renal arteriolar wall thickening [21]. Together, these observations support biological plausibility without identifying the mechanism underlying the association in our cohort.

The large size of this biopsy-proven IgAN cohort and its long follow-up allowed smoking exposure to be examined across the prolonged disease course. Separate analyses of two kidney outcomes, a primary focus on men, and complementary sensitivity analyses extend the available evidence, while accounting for the marked sex imbalance in smoking exposure and examining key modeling choices. Several limitations warrant consideration. First, the retrospective, single-center design, requirement for at least 1 year of follow-up, and complete-case analysis may introduce selection bias, including potentially informative loss to follow-up or missingness. Second, smoking was assessed only at baseline, without quantitative exposure or cessation histories, which limited dose–response assessment and inference about quitting; cessation or relapse during follow-up may have caused exposure misclassification and biased the association estimates. Third, with only 24 women reporting current or former smoking, the findings cannot be reliably generalized to women. Fourth, unavailable Oxford histopathologic lesion scores (mesangial hypercellularity, endocapillary hypercellularity, segmental glomerulosclerosis, tubular atrophy/interstitial fibrosis, and crescents) limited adjustment for disease severity. Although additional adjustment for alcohol use did not attenuate the smoking associations, the binary admission measure did not capture drinking quantity or changes during follow-up. Unmeasured diet, occupational exposures, socioeconomic circumstances, and healthcare-seeking behavior may also contribute to residual confounding. Finally, insufficient data on non-kidney deaths precluded competing-risk analysis.

In conclusion, smoking was associated with higher hazards of kidney failure and a 50% decline in eGFR among men with IgAN, with similar estimates in the full-cohort analysis. These findings are consistent with recommendations to address smoking as part of comprehensive IgAN care. However, residual confounding and limited smoking information preclude causal conclusions or inference about the kidney benefits of cessation, and the findings cannot be reliably generalized to women. Prospective studies with repeated quantitative smoking assessments, cessation timing, and more complete treatment and outcome data are needed to clarify these associations and assess changes in kidney risk after quitting.

## Data Availability

The data that support the findings of this study are available from the corresponding author, Le WB, upon reasonable request.
